# Nanodiamond surface chemistry controls assembly of polypyrrole and generation of photovoltage

**DOI:** 10.1038/s41598-020-80438-3

**Published:** 2021-01-12

**Authors:** Daria Miliaieva, Petra Matunova, Jan Cermak, Stepan Stehlik, Adrian Cernescu, Zdenek Remes, Pavla Stenclova, Martin Muller, Bohuslav Rezek

**Affiliations:** 1 Institute of Physics, Czech Academy of Sciences, Cukrovarnická 10, Prague 6, Czech Republic; 2 Faculty of Electrical Engineering, Czech Technical University in Prague, Technická 2, Prague 6, Czech Republic; 3 Attocube systems AG, Eglfinger Weg 2, 85540 Munich, Germany

**Keywords:** Energy harvesting, Electronic properties and materials, Molecular self-assembly, Organic-inorganic nanostructures

## Abstract

Nanoscale composite of detonation nanodiamond (DND) and polypyrrole (PPy) as a representative of organic light-harvesting polymers is explored for energy generation, using nanodiamond as an inorganic electron acceptor. We present a technology for the composite layer-by-layer synthesis that is suitable for solar cell fabrication. The formation, pronounced material interaction, and photovoltaic properties of DND-PPy composites are characterized down to nanoscale by atomic force microscopy, infrared spectroscopy, Kelvin probe, and electronic transport measurements. The data show that DNDs with different surface terminations (hydrogenated, oxidized, poly-functional) assemble PPy oligomers in different ways. This leads to composites with different optoelectronic properties. Tight material interaction results in significantly enhanced photovoltage and broadband (1–3.5 eV) optical absorption in DND/PPy composites compared to pristine materials. Combination of both oxygen and hydrogen functional groups on the nanodiamond surface appears to be the most favorable for the optoelectronic effects. Theoretical DFT calculations corroborate the experimental data. Test solar cells demonstrate the functionality of the concept.

## Introduction

Renewable photovoltaic (PV) energy generation provides an increasing contribution to on-grid and off-grid electricity demands in many developed and developing countries worldwide. Light energy conversion already covers a considerable part of energy requirements in many countries. Nowadays, the fabrication of PV solar cells is dominated by crystalline Si technology. Nevertheless, organic-based PV (OPV) envisioned many years ago has become a viable complementary technology to Si solar cells sector and might be Si alternative in the future. There are several reasons for this, namely (1) shorter time to return the energy used for device fabrication, (2) lower production and deployment costs, and (3) better output power under low-light or diffuse light conditions such as in hazy weather, building integration or indoor applications^1^. However, to be widely accepted, OPV still needs improvements in terms of efficiency and particularly long-term stability of operation.

Until recently, the most investigated blend for organic solar cells was a combination of poly(3-hexylthiophene) (P3HT) conjugated polymer with the fullerene (C_60_) derivatives, such as [6,6]-phenyl-C_61_-butyric acid methyl ester (PC_60_BM) and its analogue [6,6]-phenyl-C_71_-butyric acid methyl ester (PC_70_BM)^2,3^. The record fullerene-based blend efficiency of 7.4% was achieved in a blend of indene-C_70_-bisadduct (IC_70_BA) and P3HT^4^. High synthesis cost, limited optical absorption, poor bandgap tunability, morphological and photochemical instability of commonly used fullerenes motivates research of alternative materials for light-harvesting blends^5–7^.

Small molecule non-fullerene acceptors (NFA) made a break-through in OPV efficiency in the past few years due to their optical properties and electronic levels tunability as well as high thermal, photochemical and morphological stability^8,9^. Usually, synthesis of NFA involves multiple steps (6 and more) that boosts the cost immensely (e.g. 6850$/g for ITIC, one of the most representative NFAs with high power conversion efficiency)^10^.

Substituting organic acceptor in OPV with inorganic nanoparticles can bring the benefits as well, namely (1) improvement of optoelectronic stability of the solar cells; (2) enhancement of the light absorption range by changing the size or surface chemistry of the nanoparticle and consequently, its bandgap; (3) enhanced light scattering with subsequently enhanced light absorption^11,12^; (4) enhancement of the light-harvesting since the rate of the photoinduced charges transfer in the inorganic material is of a picosecond order and faster than their recombination rate; (5) morphology of the inorganic materials is tailorable to produce simultaneously efficient exciton dissociation interfaces and free charge-transporting pathways^13,14^; (6) inexpensive fabrication cost. In this respect, the different forms of carbon have been under investigation for the role of electron acceptor in OPV^15–17^. Nanodiamonds (ND) provide many unique qualities that are highly advantageous for energy conversion applications. Since covalent chemistry works similarly on NDs as for organics, various chemical moieties can be thus grafted on NDs surfaces^18^. Tunable ND surface chemistry results in tunable surface work function^19^ and in controllable interaction between NDs and surrounding materials or environment^20^. The colloidal chemistry of NDs is also well established. It enables the mixing of NDs with polymers without clustering or segregation, which is one of the crucial problems in the development of organic PV cells^21^. Except for specific surface reactions, NDs and their structural, as well as electronic properties, are very stable under various conditions for a long time^22^. The NDs are also available in large quantities at reasonable prices (0.1 $/g). Diamond nanoparticles are merely carbon, and they are completely non-toxic in the environment and organisms^23,24^. As a waste, they can be stored indefinitely or burnt into CO_2_ and they do not need any specific disposal procedures. These features enable possible large-scale use of NDs without problems.

Nanodiamond appears to be a versatile material for energy conversion. Upon mixing with C_60,_ it acts as an electron donor^25,26^. Meanwhile, bonded to P3HT^27,28^ and or porphyrin derivative^29^, nanodiamond acts as an electron acceptor. Electron-accepting properties were reported also for bulk diamond bonded with polypyrrole (PPy)^30,31^. PPy is a well-investigated organic polymer. It absorbs visible light^32^, shows good conductivity upon oxidation^33^ and can be obtained by various fabrication techniques^34–40^. Optoelectronic properties of PPy can be tuned by oxidation/reduction of the polymer and by using different electrolytes in the polymerization solution^41^. By increasing the oxidation state of PPy the number of positively charged units (polarons and bipolarons) in the polymer chain increases^41–44^ which introduces energy levels (bands in case of pronounced oxidation) into PPy bandgap^45^. This enhances PPy light absorption in the infrared region.

Therefore, in this work we explore merging NDs with PPy. We present a novel fabrication technology and optoelectronic characterization of nanocomposites based on detonation nanodiamonds (DNDs) and PPy. We reveal a tight nanoscale interaction between DNDs and PPy in the composites, which leads to enhanced optical absorption and more efficient charge generation as shown by various characterization methods and test solar cells. We provide theoretical calculations of the structure and electronic properties of DND/PPy composites, which are in good agreement with the experimental data and which help elucidate the mechanism of exciton dissociation in DND/PPy composite.

## Materials and methods

### Materials

Original DNDs were manufactured by Lingyun Granda Nano (China) and distributed by New Metals and Chemicals (Japan). Fourier-transform infrared spectroscopy (FTIR) of the purchased DNDs showed in the as-received state pronounced peaks at 2800–3000 cm^−1^ that correspond to C–H surface groups as well as peaks at 1800 cm^−1^ due to carbonyl C=O groups. Therefore, to emphasize the presence of multiple types of surface moieties (related to both oxygen and hydrogen), we will refer to the as-received DNDs as polyfunctional-DNDs (poly-DND) in the further text. The average size of poly-DND claimed by the manufacturer is around 5 nm. Zeta potential of polyfunctional-DNDs dispersed in water is positive, + 41 mV (measured by Dynamic Light Scattering using Malvern Zetasizer Nano ZS). The presence of sp^2^ carbon atoms on the poly-DND surface is indicated by 1630 cm^−1^ band in Raman spectrum (see Figure S1 in the Supplementary Information). Oxidized DNDs (O-DND) were obtained from the polyfunctional-DNDs by annealing in air at 450 °C for 30 min. Such oxidation conditions do not lead to poly-DND size reduction^46^, just surface functional groups are transformed, namely CH into CO groups. Zeta potential of such air-annealed DNDs is -39 mV. The air annealing leads to the decrease of sp^2^ carbon content on O-DND compared to poly-DND surface (Figure S1). Hydrogenated DNDs (H-DND) were obtained from O-DNDs by annealing in hydrogen at 600 °C for 6 h^47^. Our DLS measurements reveal H-DND size to be around 3 nm. Zeta potential of H-DND is + 40 mV, thus similar to poly-DNDs, yet the FTIR spectra are considerably different (see Figure S2 in the Supplementary Information). Hydrogenation at 600 °C leads to additional surface graphitization which estimates itself in the most pronounced and redshifted peak (1600 cm^−1^) of sp^2^ carbon in Raman spectrum (Figure S1).

### Nanocomposite fabrication

**Figure 1:**
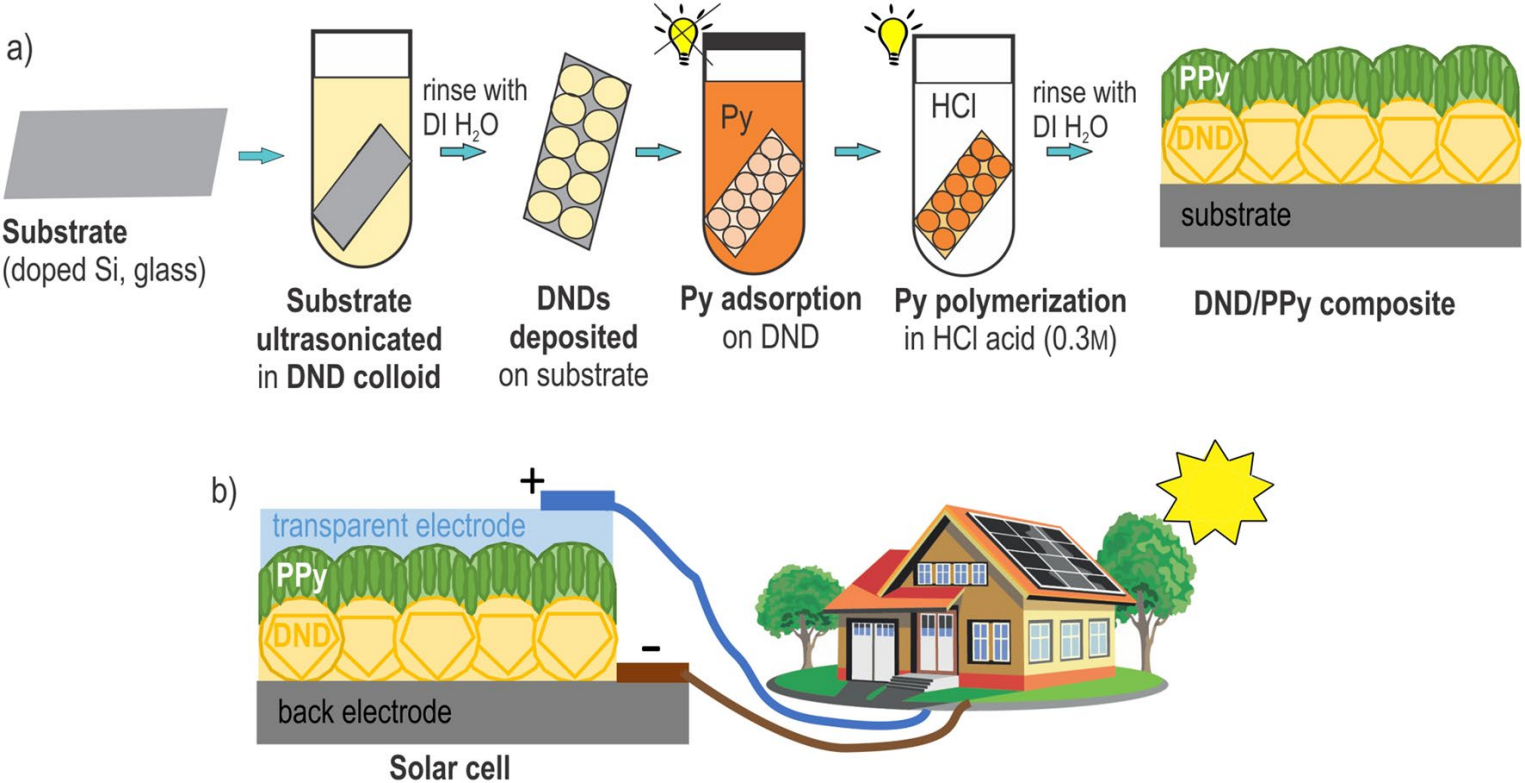
Scheme of (**a**) the fabrication process of DND/PPy composite and (**b**) test solar cell with embedded composite as an active material.

Figure 1 shows a scheme of the layer-by-layer technological procedure developed for the preparation of DND/PPy nanocomposite layers on substrates. The substrates (silicon or glass) were nucleated with DND particles by sonication for 10 min in a colloidal solution of primary DND particles (prepared according to the established protocol^46^) and then they were rinsed with deionized water. Nucleation of the substrates by DND resulted in 10% and 40% monolayer coverage of the substrates by poly-DND or H-DND respectively^47^. Direct nucleation of the substrates by O-DND, in the same way, was complicated due to electrostatic repulsion between oxidized DNDs and oxidized silicon or glass surface. Thus, to get similar Si and glass nucleation with O-DNDs, we nucleated the substrate with poly-DND at first and then annealed the substrates in the air at 450 °C for 30 min. The procedure results in 8% coverage of the substrate by O-DND. The morphology of DND monolayers was characterized by AFM (Figure S3 in the Supplementary Information).

Substrates with a low density of DND agglomerates (about 30 agglomerates per 1 µm^2^) were prepared to obtain nanoscale spectroscopic data on the particles and the surrounding substrate separately. For this purpose, DND powder was sonicated for 1 h using an ultrasound horn (Hielscher) at 200 W, then the solution was centrifuged at 14,000 rpm (13,124×*g*) for 25 min to get rid of micrometre-sized DND clusters. Then the supernatant was poured into the clean test tubes and further centrifuged for 30 min. The sediment after 30 min of centrifugation was re-dispersed in 1 ml of water and further diluted 20 times. The procedure allowed us to separate agglomerates with the sizes on average larger than the SNOM-IR tip diameter ~ 20 nm. The sizes and spatial distribution were estimated from SEM images (see Figure S4 and Figure S5 in the Supplementary Information).

After nucleation with DNDs, the substrates were soaked in pyrrole (Py) monomer for 1 h to let the Py monomers adsorb (see Fig. 1). Then the substrate with adsorbed Py was immersed into HCl acid (0.3 M) for 16 h under indoor illumination to promote Py polymerization. The substrate was then taken out of HCl, rinsed with deionized water, and dried with N_2_. Hydrochloric acid (HCl, 37%) and pyrrole (98%) for polypyrrole synthesis were purchased from Sigma-Aldrich (USA) and used in the as-received state. Substrates processed in the same way, but without DNDs, were used as PPy reference substrates. Substrates nucleated with DND particles without further Py processing were used as DND reference substrates. PPy layer morphology and thickness were again investigated by AFM.

To increase the thickness of the DND layer the spin-coating method was used instead of treatment in an ultrasonic bath and different spin-coating procedures were tested. The spin-coating procedure, which provided DND layers of the most uniform thickness with the employed colloid concentration (38 ± 3 nm, see Figure S6 in the Supplementary Information), was chosen for the sample preparation, namely 100 μL of the DND colloidal solution was drop-casted on 1 cm^2^ sample and rotated at 1000 rpm for 15 s, with subsequent increase of the speed to 4000 rpm and rotating at this speed for 1 min. The same Py grafting procedure used for the nucleated monolayer of DNDs was also employed for the spin-coated DND layers. The thickness of PPy on the spin-coated layers was determined as the difference between the average composite layer thickness in secondary electron and backscattered electron mode of Scanning Electron Microscope, where nanodiamond layer looks as the brightest stripe and PPy is a black layer on top of DND (see Figure S7 in the Supplementary Information).

### Test solar cell (SC) fabrication

For the preparation of the test solar cell (Fig. 1b), a back electrode was covered with DND/PPy composite as an active material, and a counter electrode was sputtered on top. Different materials were tested as back electrodes: metals (Au, Al), glass with ITO film (with and without poly(3,4-ethylenedioxythiophene) polystyrene sulfonate, PEDOT:PSS), silicon (Si) with different doping (p, p++, n, n++). As the top electrode, semitransparent Al and Au (18 nm thickness) and ITO (140 nm thickness) stripes of approximate 0.05 cm^2^ area were prepared. Different thicknesses of DND layers also tested.

### Characterization techniques

Atomic force microscopy (AFM, NTMDT Ntegra) was used in the tapping mode (Si cantilevers with 75 kHz resonance frequency) to characterize the morphology and thickness of the reference poly-DND, H-DND and O-DND nucleated substrates as well as the substrates after the composite formation. Part of the particles on each sample was carefully removed by a thin wood stick. The samples were scanned perpendicular to the interface between the exposed and nucleated part of substrates. Height of the bare substrate was taken as the zero height. Height of each grain on the samples was determined by using a watershed algorithm^48^ relative to the exposed substrate. Mean heights of composite and reference DNDs particles were calculated. The PPy layer thickness on DNDs was determined by the formula in^49^.

Scanning electron microscopy (FE-SEM Tescan MAIA3) was used as a complementary tool to characterize surface morphology. Field-emission gun operating at 10 kV and in-beam secondary electron detector was used.

Dynamic Light Scattering (DLS) measurements of colloidal solutions were performed in order to estimate the size and colloidal stability (zeta potential) of DNDs. Zetasizer Nano ZS from Malvern Instrument Ltd. equipped with a helium–neon laser (633 nm) and the scattering angle 173° was used for the measurement. Scattering intensity/size distribution data was recalculated to the number/size distribution curve using Mie theory. Each curve was plotted after accumulation of scattering intensity from 10 runs. The size of the particles was estimated as the average from 3 number/size distribution curves. Zeta potential distribution curve was calculated automatically in program from Henry equation after estimation of the electrophoretic mobilities of the particles under applied voltage in the special cell with the electrodes. Zeta potential value was calculated as the average from 3 distribution curves.

Raman spectroscopy of the nanodiamonds was done in Renishaw InVia micro-spectrometer equipped with 442 nm excitation HeCd laser using 100 × objective (numerical aperture 0.9). The used laser power was approximately 2 mW. The Raman signal from each DND sample was accumulated for 500 s and spectra from three different spots were added. Due to a photoluminescence background the Raman spectra were baseline corrected and normalized to the diamond peak.

Formation of the DND/PPy composites was characterized by infrared spectroscopy: macroscopically by grazing angle reflectance Fourier-transform infrared spectroscopy (GAR-FTIR) and microscopically by scanning near-field optical microscopy in the infrared region (SNOM-IR). GAR-FTIR spectra were measured using N_2_-purged Thermo Nicolet 8700 spectrometer equipped with KBr beam splitter and MCT detector cooled by liquid nitrogen. Au mirrors were chosen as substrates for GAR-FTIR measurements to ensure a high enough signal from the samples. The optical absorbance was calculated in the standard absorbance units as A =  −log(R/R_0_), where R is the spectrum of analyzed material and R_0_ is the reference (background) spectrum recorded using a clean Au mirror prior to the analyte application. In all cases, the spectra represent an average of 128 scans recorded with a resolution of 4 cm^−1^. Each FTIR spectrum was normalized to the intensity of the strongest peak in the spectrum.

SNOM-IR spectra were collected with ca. 20 nm spatial resolution using a scattering-type near-field optical microscope (neaSNOM, neaspec GmbH) equipped with a broadband Difference Frequency Generation (DFG) laser source (Toptica) and an asymmetric Michelson interferometer described elsewhere^50^. PtIr tip with ca. 20 nm diameter was used to probe the sample by operating in tapping mode (tapping amplitude ca. 40 nm) at the frequency Ω of ca. 270 kHz, thus modulating the intensity of the scattered IR light at Ω and its higher harmonics. Individual spectra were recorded in ca. 3 min each, with a spectral resolution of 8.3 cm^−1^. Demodulation of the scattered light signal at a higher harmonic nΩ was used to separate the contribution of the near-field optical signal from that of the background signal^51^. Removal of the instrumental response function from the SNOM-IR spectra was done by normalization of the measured spectra to a reference Si signal. Resulting SNOM-IR Absorption and Reflectivity spectra can be directly correlated with the standard far-field IR spectra^52,53^.

Photothermal deflection spectroscopy (PDS) was used to characterize optical transmittance, reflectance and absorbance spectra in the broad spectral range from UV to near IR (250–1700 nm). The absorption coefficient (1/cm) at each wavelength was calculated from absorptance spectra and the thickness of the samples as

$$ Absorption \, coefficient = \frac{{{-}\ln \left( {1 - Absorptan ce} \right)}}{Sample \, thickness } . $$

The thickness of the PDS samples prepared on glass was evaluated by AFM. Figure S8 shows the thickness evaluation from AFM images on the DND-PPy composite samples. During the PDS measurements, the samples were immersed into transparent liquid FC72 and their absorptance spectra were normalized via absorptance spectrum of the highly absorbing black coating^54^.

Scanning Kelvin Probe (SKP, KPTechnologies) method was used to determine the surface potentials (SP) of the samples in the dark and under illumination. The light source was the halogen lamp (maximum power 250 W) with the broad emission spectrum in the visible region 375–800 nm with the maximum at ~ 650 nm. Subsequently, surface photovoltages were calculated. SKP advantage compared to Kelvin Probe Force microscopy is the possibility of “absolute” dark conditions of measurements as no laser is required for probe position detection in SKP. The scanning of each sample was conducted with a golden tip of 2 mm in diameter at 25 different points. The value of the surface potential for the specified sample was measured at 25 points in the dark and at the same points under illumination. The original data of SP measurements is provided in Figure S9. For some materials like O-DND/PPy, H-DND/PPy and neat O-DND the systematic error of the measurement is seen in the slope of SP curves. To eliminate this error of the measurements, it was decided to calculate the SPV at each point of the measurements as the difference between SP under illumination and SP in the dark at the point; then average the SPVs from all scanned points to obtain the final SPV value for the particular sample.

Current–voltage (IV) characteristics were measured by needle probes in a measurement setup using a source-measure unit (Keithley). One of the microelectrodes was in contact with a steel carrier disk. The solar cell back electrode was fixed to the steel disk by a gold paste. The other microelectrode is in contact with the solar cell top electrode through a silver paste. The current was measured in a voltage range ± 0.4 V with the sweep rate 50 mV/s. IV curves were measured in the dark and under AM 1.5 solar simulator illumination (light intensity 1000 W/m^2^). Power conversion efficiency (PCE) of the cell was calculated as

$$ PCE\% = \frac{{P_{\max } \times 100\% }}{{P_{incident} }} , $$

where *P*_*incident*_ = *Light intensity of solar simulator* × *Area under illumination* is the power of incident light and P_max_ is the maximum output power of solar cell which is defined by the maximum product of input voltage and corresponding current. The area under illumination is equal to the area of the top electrode.

### Theoretical computing procedures

The first-principles density functional theory (DFT) method implemented in Gaussian 09 software^55^ was used for optimizations of all the structures to obtain the ground state configurations. B3LYP hybrid functional^56,57^, one of the most widely used functionals in computations of organic molecules, was employed together with the 6-31G(d) basis set. Modelling whole ND with experimentally accessible size starting at 1.4 nm^58^ would be computationally unfeasible at this level of theory. Therefore, we used (111) and (100) surface slabs consisting of 3 C double layers of 6 × 6 atoms, exceptionally 5 × 6 atoms if structurally needed. The slabs represent a corner of ND, hence always three “outer” surfaces of ND are functionalized with surface groups, and the remaining three “inner” surfaces representing the inner cut planes are saturated with H atoms to keep the sp^3^ hybridization of neighbouring carbon atoms. These H atoms were fixed during further optimization. Further details on structure optimization and energy levels calculations can be found in^59,60^.

## Results and discussion

**Figure 2:**
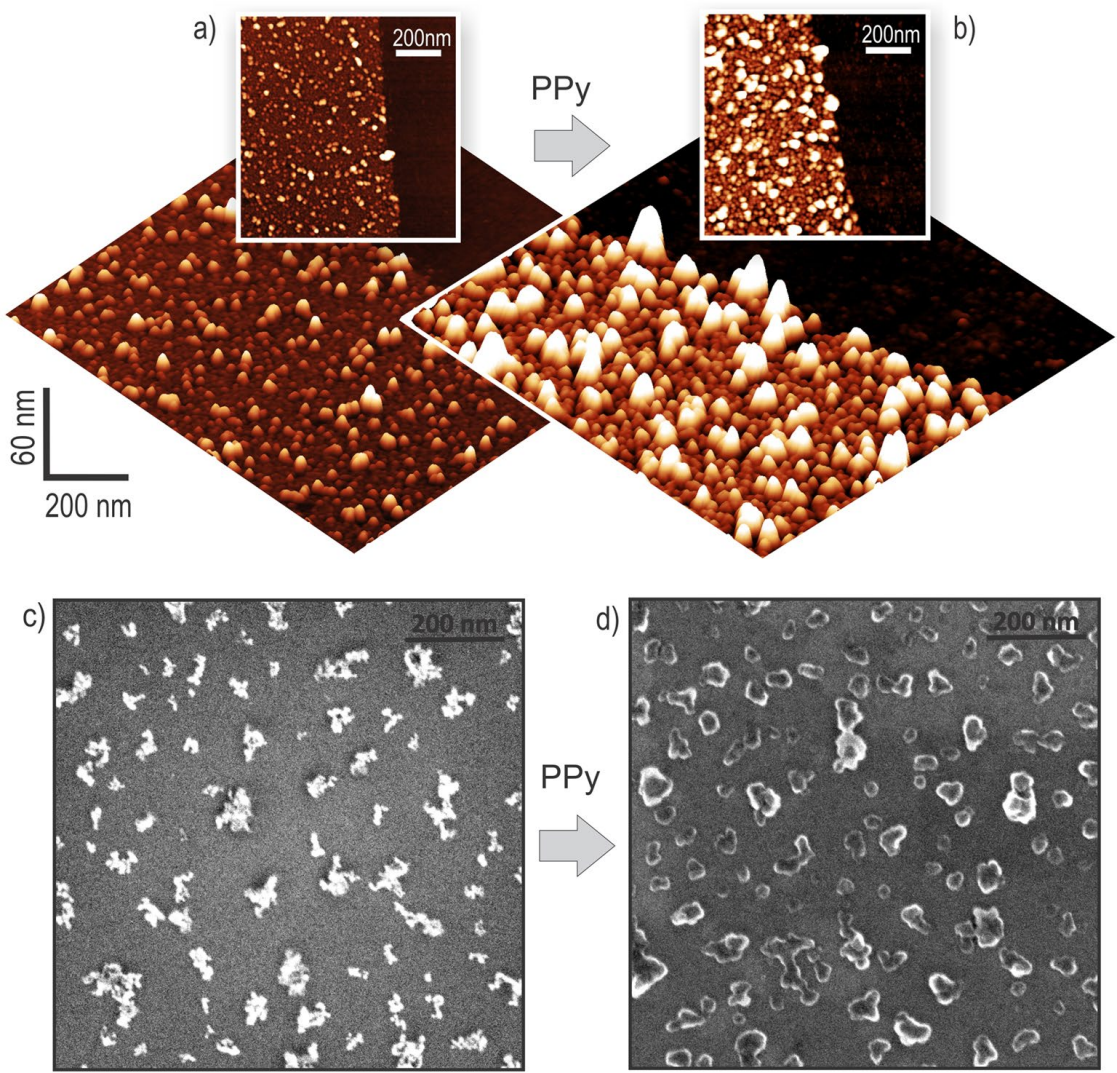
AFM images of (**a**) pristine H-DND layer and (**b**) H-DND/PPy composite layer. SEM images of intentionally scattered poly-DND agglomerates (**c**) before and (**d**) after modification by PPy.

Figure 2a, b show topography images of DND monolayer before and after Py processing obtained by AFM. Planar images show particle distribution and height differences across the surface. 3D AFM images visualize overall size enlargement after processing of the DNDs with Py. They suggest encapsulation of DND by PPy. Figure 2c, d show SEM images of intentionally prepared scattered DND clusters on Si substrate before and after Py processing. The SEM images corroborate observations by AFM that the DNDs are conformally encapsulated by PPy after the processing.

As can be seen from the AFM images, already pristine DND particles form aggregates of different sizes on the substrate (Fig. 2a). To understand how surface chemistry influences DNDs interaction with the PPy polymer we selected the sizes that correspond to primary particles (for poly-DND and O-DND it is 4.5–5.0 nm, and for H-DND it is 3 nm) or 2-particle aggregates. Larger aggregates introduce uncertainties in the PPy thickness interpretation due to, e.g., different surface chemistry of aggregates compared to the primary particles. Note that primary particles were needed only for reliable PPy thickness determination. For physico-chemical characterization by Raman and FTIR spectroscopies it does not matter if there are primary or aggregated particles. For optoelectronic properties the agglomerated particles can form a continuous network for charge transfer from the nanodiamond-polymer interface to the electrode, which may make agglomerated nanoparticles more beneficial in optoelectronic applications. Evaluated mean particle sizes are summarized in Table 1. Mean sizes of poly-DND (4.8 ± 0.2 nm) and O-DND (5.1 ± 0.4 nm) are similar. This confirms that the annealing does not reduce the particle size but modifies the surface groups only. H-DND mean size is somewhat smaller, 2.9 ± 0.1 nm, as expected from the hydrogenation process^33^.

**Table 1:** Mean height of DND particles and the composites established from AFM topography data and calculated PPy thicknesses.

Sample name	Mean Particle Height, nm	PPy thickness, nm
Poly-DND	4.8 ± 0.2	4.2 ± 0.4
Poly-DND/PPy	9.0 ± 0.3	
H-DND	2.9 ± 0.1	3.8 ± 0.1
H-DND/PPy	6.7 ± 0.1	
O-DND	5.1 ± 0.4	1.4 ± 0.7
O-DND/PPy	6.5 ± 0.6	

After the pyrrole processing, there is an enlargement in particle height (see Table 1) which depends on the initial particle surface chemistry. The thickest PPy layer is formed on poly-DND (4.2 ± 0.4 nm), and the minimum PPy thickness is observed on O-DND (1.4 ± 0.7 nm). PPy layer on H-DND (3.8 ± 0.1 nm) is of similar thickness as on poly-DND. PPy thicknesses on O-DND and poly-DND appeared to be smaller than estimated in our previous work^49^. This might be due to the more accurate particle height evaluation procedure in this work by grain analysis using the watershed method (see the Experimental section) compared to particle height estimation via roughness analysis in^49^, where mean particle height was determined as the difference between the roughness of nucleated and exposed parts of a substrate. However, different particle height evaluation methods lead to the same trend in PPy thickness which is maximum on poly-DND and 2–3 times less on O-DND. Because of the same Py polymerization conditions for all types of DND particles, different PPy thickness on poly-, H- and O-DND, as was discussed in^49^, can be explained by the different PPy chains arrangement relative to the different types of DNDs. The different PPy arrangement might be caused by the different bonding nature between PPy and DNDs of different surface terminations, which is the matter of our further investigation by spectroscopic techniques.

**Figure 3:**
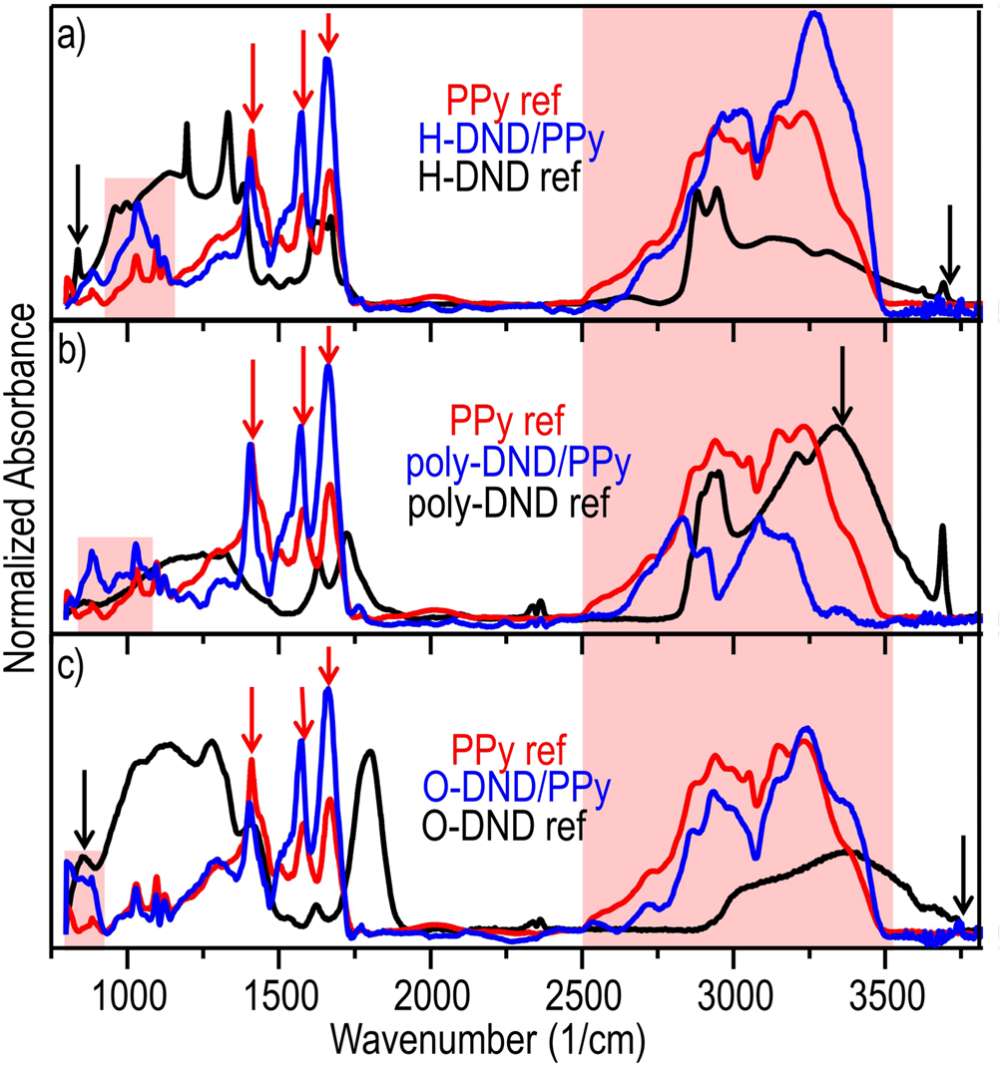
FTIR spectra of (**a**) H-DND/PPy (**b**) poly-DND/PPy and (**c**) O-DND/PPy composites with the reference DNDs and PPy. The remaining features of the pristine materials in the spectra of composites are indicated by arrows, namely pristine DND (black arrows) and PPy (red arrows).

Interaction of DND and PPy in the composites was thus characterized by the IR spectroscopic techniques. Figure 3 shows FTIR spectra of DND/PPy, pristine DNDs and pristine PPy directly on layered samples (unlike in our prior work where bulk material was analysed). The spectra indicate the formation of the nanocomposites. The remaining features of the pristine materials can be found in the spectra of the composites and are indicated by arrows, namely pristine DNDs (black arrows) and PPy (red arrows).

At the same time, some of the characteristic vibrations of DNDs are quenched by interaction with PPy. The band of C–H vibrations at 2800–3000 cm^−1^, is clearly pronounced in the initial H-DNDs and poly-DNDs spectra. It is missing in poly-DND/PPy composite spectrum. It seems to be also missing in H-DND/PPy composite spectrum, yet some C-H vibrations may be hidden in the band of O–H∙∙∙Cl and N–H∙∙∙Cl vibrations at 2800–3000 cm^−1^^61,62^. The peak of carbonyl group, which is present in the reference O-DND spectrum at 1800 cm^−1^ is completely missing in the O-DND/PPy composite spectrum. The regions of 800–1100 and 2250–3770 cm^−1^ (red background) reveal the new features in the DNDs/PPy composite compared to the pristine components. Moreover, the difference between the composites' spectra in these regions suggests the different type of interaction between PPy and DND of different surface terminations. The peak at 1034 cm^−1^ of poly-DND/PPy composite has a distinct shoulder at 970 cm^−1^, which is absent in the PPy reference spectrum. This feature is attributed to C–C out-of-plane deformations of PPy ring^63,64^. In H-DND/PPy, the shape of 1034 cm^−1^ peak differs from the one of PPy reference, it is broadened and with the shoulder of C–C out of plane vibrations as in poly-DND/PPy. In O-DND/PPy, the region in 800–930 cm^−1^ seems to be a superposition of DND (850 cm^−1^ peak can be attributed to C–Cl in diamond particle^65^, and thus it indicates possible partial chlorination of O-DND surface after interaction with HCl solution) and PPy vibrations (880 cm^−1^ peak can be attributed to C-H out of PPy ring plane deformations^63^).

The range in 2250–3770 cm^−1^ represents a complex mixture of OH, NH and O–H∙∙∙Cl and N–H∙∙∙Cl vibrations^65^. There is a noticeable redshift of the poly-DND/PPy bands in 2250–3770 cm^−1^ region compared to other composites and PPy ref. The shape of poly-DND/PPy spectrum in this region differs from the spectrum shape of the other materials. It might be due to the largest amount of O–H bonds on the poly-DND, surface compared to H-DND and O-DND (carbonyl and carboxyl groups dominate in O-DNDs) particles. Thus, O–H∙∙∙Cl dominates in poly-DND/PPy in the 2250–3770 cm^−1^ region while spectra of the other composites in this range are dominated by N–H∙∙∙Cl or NH vibrations and they are blue-shifted.

FTIR, measuring the averaged macroscopic signal from the layers, does not enable to distinguish between the signal from PPy on the particles and PPy on the substrate of the same sample. To overcome this limitation of FTIR and further elucidate the differences in the composites structure, we performed SNOM-IR spectroscopy with a nanoscale resolution on the DND agglomerates. Figure S4 shows the uniform distribution of the isolated DND agglomerates across the surface by SEM and their height morphology by AFM. It shows that the agglomerates are indeed larger than SNOM-IR tip diameter (20 nm) and that they are separated enough for SNOM-IR measurements on individual agglomerates. SEM images in Fig. 2 (and in Figure S5 for a complete overview) show the DND agglomerates before and after pyrrole processing. They evidence that the DNDs agglomerates are indeed coated by PPy.

**Figure 4:**
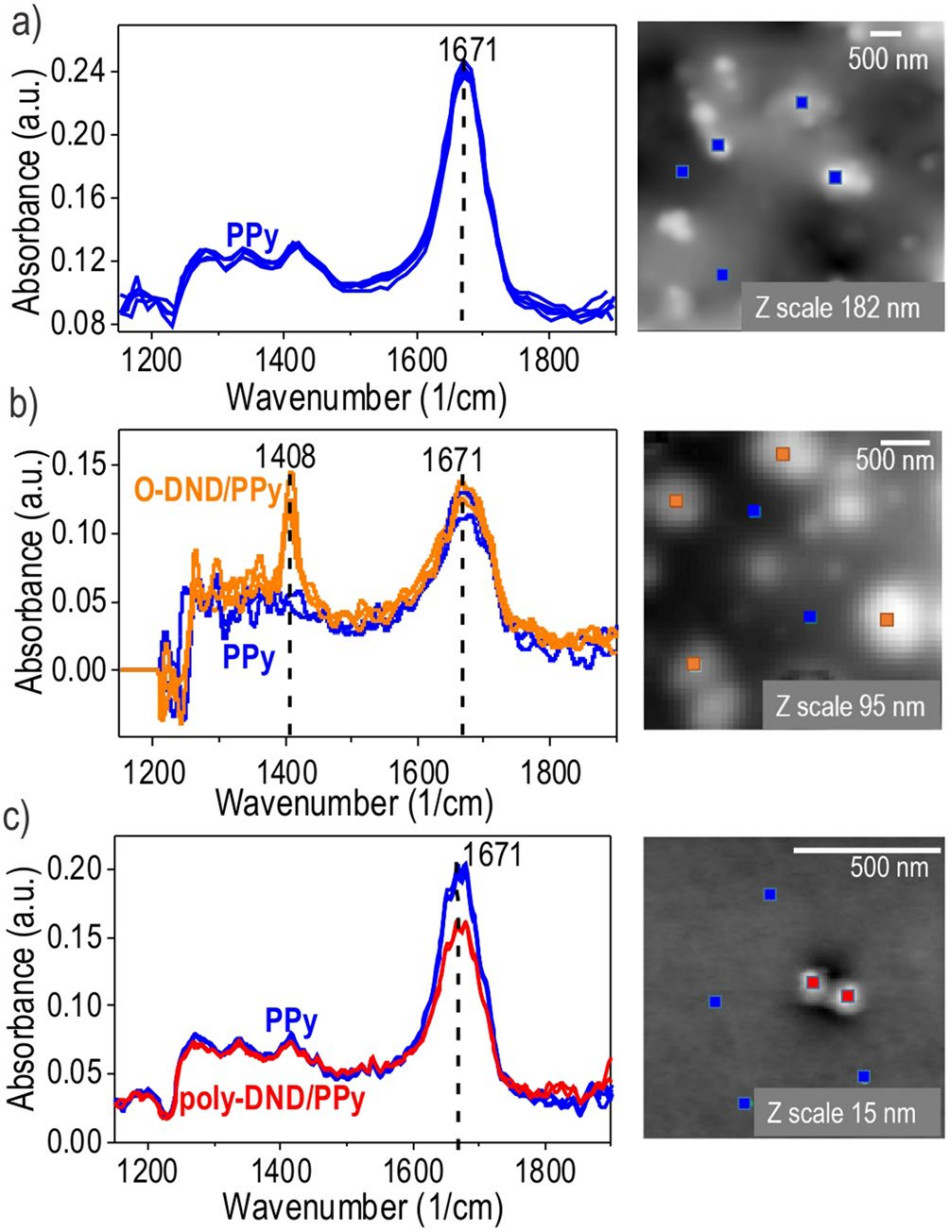
AFM maps with indicated locations of SNOM-IR spectra of (**a**) PPy reference (**b**) O-DND/PPy (**c**) poly-DND/PPy. The lateral size of the particles’ and PPy agglomerates could be deduced from the scale bars on AFM images. Characteristic PPy peaks at 1671 (N–H vibration) and 1408 (C–N vibration) cm^−1^ are indicated at SNOM-IR spectra.

In Fig. 4, SNOM-IR spectra of the poly-DND/PPy, O-DND/PPy and PPy reference are presented. Topography images of the samples at the same place are shown as well. The spectra of given colour correspond to the place on the topography image marked by the rectangle of the same colour. Figure 4a shows PPy reference spectra at 5 different locations on the substrate, including both plains and hillocks, which all match well with each other.

Figure 4b shows the spectra of PPy on O-DND particles (orange line) and their surroundings (blue line). Several spectra taken on O-DND/PPy particles match with each other and reveal a distinct peak at 1408 cm^−1^ not found in the spectra of the surroundings. Figure 4c shows spectra taken on the poly-DND/PPy particles (red lines) and on the substrate (blue line). The spectra are well reproducible. Spectra taken on the substrate differ from the ones on poly-DND/PPy particles by the more pronounced intensity of the peak at 1671 cm^−1^, while in the rest of the spectral region the spectra match well.

A new peak at 1408 cm^−1^ in O-DND/PPy compared to PPy reference can be attributed to C–N vibrations which became asymmetrical (due to electrostatic interactions of O-DND and PPy) and subsequently visible in SNOM-IR spectrum. The peak at 1671 cm^−1^ presented in all SNOM-IR spectra is most likely attributed to N–H vibrations. The intensity of the peak does not depend on the thickness of PPy layer (the spectra on PPy references taken on PPy micron-size agglomerate and PPy thin film of about 20 nm thickness are comparable, see Fig. 4a). Interaction of PPy with O-DND does not influence 1671 cm^−1^ peak intensity since it can be of the same electrostatic nature as the interaction between PPy and silicon (with native silicon oxide) substrate in PPy reference. Thus, the difference in 1671 cm^−1^ peak intensity in poly-DND/PPy composite suggests different bonding nature with a different arrangement of PPy on Si substrate (e.g., PPy overlaying the substrate) and on a poly-DND particle (e.g., upright alignment of PPy chain relatively to poly-DND as was discussed in^49^). In this way, differently aligned PPy will interact in a different way with SNOM-IR polarized light resulting in different peak intensities. The possibility of revealing the arrangement of the molecules by the different intensity of SNOM-IR response signal has already been shown^66^ and is revealed again in this work. Thus, SNOM-IR clearly shows tight interaction between DNDs and PPy, confirming O-DND/PPy and poly-DND/PPy composites formation. Also, based on SNOM-IR results different types of bonding with subsequent different PPy arrangement relatively to O-DND and poly-DND is confirmed.

**Figure 5:**
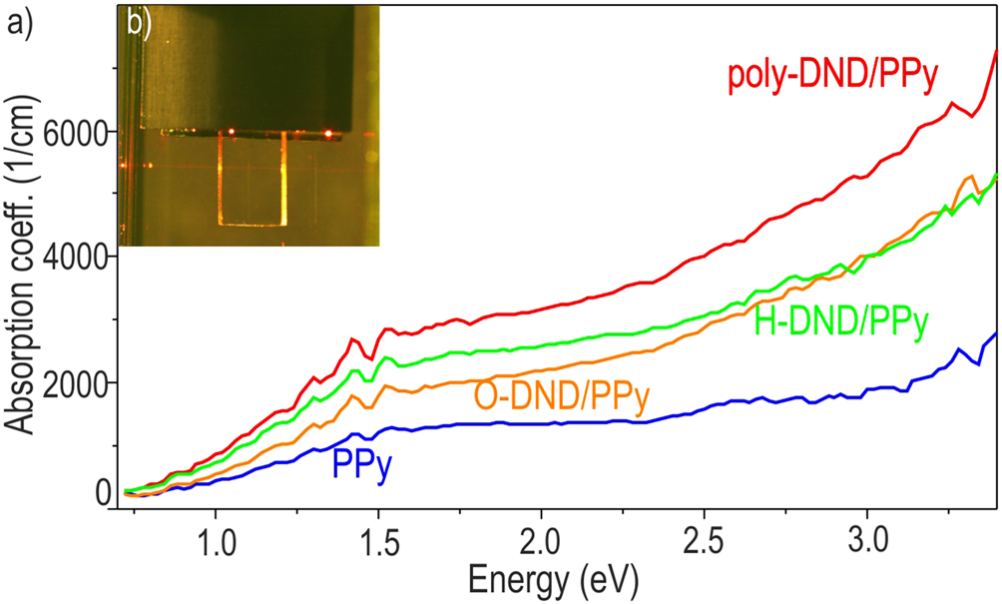
(**a**) Absorption coefficient spectra of composites and PPy reference (**b**) poly-DND/PPy sample during PDS measurement.

PDS spectra of neat PPy, as well as DND/PPy composites presented in Fig. 5, reveal optical absorption in a broad visible spectral range. Poly-DND/PPy composite absorbs the most while neat PPy absorbs the least. O-DND/PPy and H-DND/PPy composites have an absorption coefficient similar to each other, and both are well below the absorption coefficient of poly-DND/PPy composite. Although superior optical absorption of poly-DND/PPy composite seems obvious, various factors should be considered before making such a conclusion.

H- and poly-DND particles carry a larger amount of light-absorbing polymer since PPy thickness on the substrates nucleated with these DNDs is higher compared to PPy thickness on the bare substrate (see Table 1 and Figure S8 in the Supplementary information). The absorption coefficient is independent of material thickness. Nevertheless, PPy layers of different thicknesses might have PPy chains of different conjugation lengths, thus different optical properties. However, PPy thickness on O-DND is smaller than the neat PPy layer, and yet the O-DND/PPy has a larger absorption coefficient in the whole spectrum. There is also different PPy thickness on H-DND and O-DND and yet the absorption coefficient spectra are similar. Contrary, the PPy thickness on H-DND and poly-DND is similar and yet the absorption coefficient spectra differ considerably. Thus, the difference in PPy thickness in the materials in our work does not affect PPy conjugation length and consequently optical properties. Also, the pronounced difference in absorption in the composites cannot be only due to scattering effects, though as observed for instance, for Si quantum dots^11^. Otherwise, poly-DND/PPy and O-DND/PPy absorption would be similar due to the similar DND density of these samples; also H-DND/PPy would have more pronounced absorption coefficient since there are 4 times more scattering centres on the substrates nucleated with H-DND compared to O-DND and poly-DND nucleated substrates (surface coverage of Si by H-DND is ~ 40%, by poly-DND ~ 10%, by O-DND ~ 8%).

Another factor of the different absorption coefficients of the composites and PPy reference can be different electronic states of PPy itself. Although the synthesis process (and chemicals) was the same for all samples, the PPy could be different due to DND effects on the synthesis. Two additional energy levels are introduced into PPy bandgap at mild PPy oxidation, which corresponds to bonding and anti-bonding polaron states^67^. With a large extent of PPy oxidation, the quantity of intra-bandgap levels increases and they merge into bands (bipolaron bonding and anti-bonding bands). As a result, PPy oxidation promotes absorption in the infrared region and broadening of the bands in the absorption spectrum due to more possibilities of charge transfer between intra-bandgap bands. Thus, more pronounced absorption in composites compared to pristine PPy can be explained by a larger extent of PPy oxidation in composites due to charge exchange between PPy and DNDs. Poly-DND/PPy with maximum absorption compared to O-DND/PPy and H-DND/PPy might be consisted of PPy in the largest oxidation state due to the most pronounced charge exchange between poly-DND and PPy.

The last but not least factor behind the enhanced absorption coefficient of the nanocomposites may be the favorable optoelectronic properties arising at the DND/PPy junction. Theoretical computing (described further below) indicated large variations of energetic bandgap (0.3–3.5 eV) at the junction of DND/PPy depending on facet crystallographic orientation, surface chemistry and way of PPy attachment. In our opinion, it is probably the dominating effect behind the enhanced absorption coefficient as it is well correlated with photovoltage measurements by SKP.

**Figure 6:**
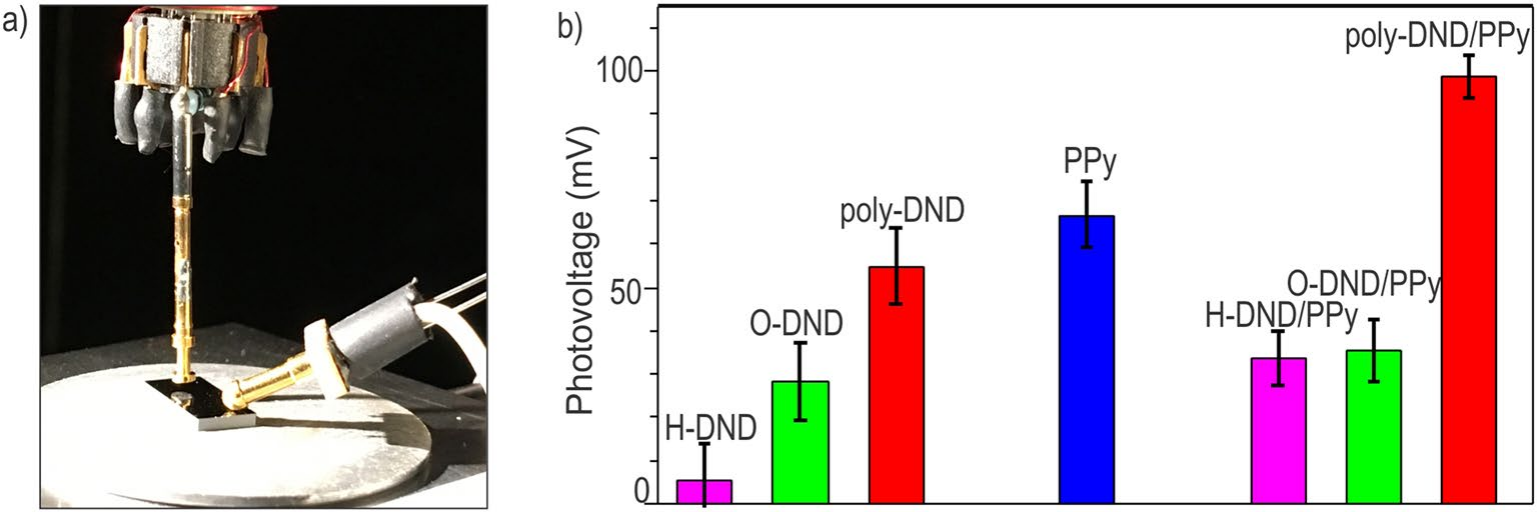
(**a**) Scanning Kelvin probe setup. (**b**) Surface photovoltages (with error bars) of DND/PPy composites, DND and PPy references.

The photograph of the SKP setup is shown in Fig. 6a. Each sample was contacted sideways by a ball-shaped electrode. SKP probing tip is above the sample. Illumination, when it is necessary, is introduced under an angle from the side. Surface potentials of poly-DND/PPy, O-DND/PPy and H-DND/PPy composites, their references and bare substrates were measured in the dark and under illumination. The original data of SP measurements are provided in Figure S9. Surface photovoltages of the samples were then calculated. The SPV values for the reference and composite samples are summarized in Table S1 and plotted in Fig. 6b.

Maximum photovoltage is observed on poly-DND/PPy composite (99 ± 5 mV) compared to O-DND/PPy (35 ± 7 mV) and H-DND/PPy (34 ± 6 mV). SPV of poly-DND/PPy is also larger compared to SPV of pristine PPy (67 ± 8 mV) and pristine poly-DNDs (55 ± 9 mV). H-DND reference sample shows negligible SPV (6 ± 8 mV), and O-DND reference has SPV values of 28 ± 9 mV. As was shown by Matunova et al.^60^, amorphization of nanodiamond surface reduces the bandgap without dependance on the surface termination. H-DND is expected to have smaller bandgap compared to O-DND due to more prounounced surface graphitization after hydrogen annealing (Figure S1). Recombination of light-harvested charges in H-DND is thus more probable than in O-DND, consequently, SPV is decreased in H-DND compared to O-DND. The largest positive surface photovoltage detected in poly-DND/PPy compared to all other samples might indicate the largest extent of PPy oxidation by poly-DNDs via withdrawing electrons from PPy chain (in agreement with the PDS spectra interpretation above). Moreover, O-DND/PPy and H-DND/PPy have similar surface photovoltages (SPV) considering the standard deviations depicted in Fig. 6. It is intriguing as the PPy assembly on H- or O-DND is different (see Table 1). Contrary, poly-DND/PPy composite exhibits SPV significantly higher compared to H-DND/PPy and O-DND/PPy, even considering the standard deviation. This points out clearly that each of the functional groups on the surface of DND, namely hydrogen-containing (C-H groups on the diamond surface create reactive oxygen species under illumination^68^ which can initiate pyrrole cation radical formation, the first polymerization step) and oxygen-containing (adsorption places for Py/PPy chains^69^) plays its specific role in poly-DND/PPy composite formation and optoelectronic performance and their combination is essential for providing enhanced SPV and light absorption in the visible region. As seen from Raman spectra (Figure S1) normalized to diamond peak (1323 cm^−1^) the content of sp^2^ phase (band in 1600 cm^−1^ region) increases after hydrogenation and diminishes after annealing in air. The trend of the sp^2^ content in Raman does not correspond to SPV trend in DND ref. Thus, the sp^2^ carbon phase is not the determining factor for SPV effect. Its role may still be in additional photo-senzitization and facilitation of the transport of light-generated charges as suggested in the work of Kokal et al.^70^ for carbon dots in TiO_2_/CdS photoanode assembly. Note also that SPV of the composites cannot be due to the substrate because the annealed p-Si has SPV of the opposite sign (− 87 ± 29 mV) compared to the nanocomposites and other reference samples.

**Figure 7:**
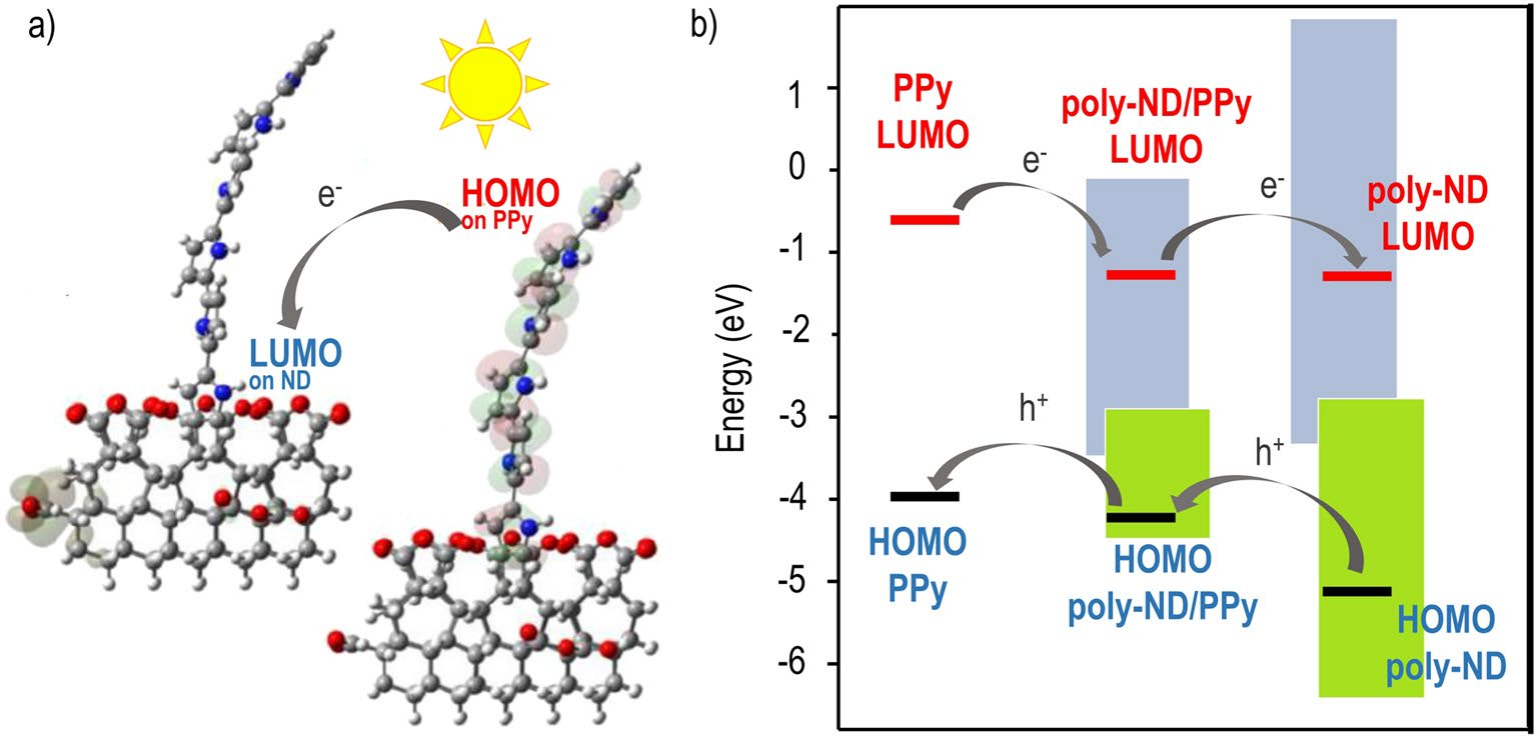
(**a**) Example of spatial separation of PPy HOMO and ND LUMO. Based on DFT calculations. (**b**) Scheme of HOMO and LUMO energetic levels composite of separate materials and their interface with the most probable charge transfer due to level alignment.

Theoretical calculations support the experimental results and help us propose a photovoltage generation mechanism in the DND/PPy composites. In the model configuration of poly-DND/PPy composites, where PPy chain grafted covalently to ND and surrounded by oxygen-containing groups, it was observed that HOMO is fully localized on PPy and LUMO fully localized on ND (Fig. 7a). In addition, for various oxygen moieties in the poly-DND/PPy models, the energetic levels of PPy, ND and ND-PPy interface are aligned in such a way that it is energetically beneficial for the electrons to be transferred from PPy into ND (Fig. 7b).

Thus, the maximum and positive SPV observed in the poly-DND/PPy composite is theoretically explained. The calculations also elucidate the role of hydrogen- and oxygen-containing groups on the poly-DND surface, namely: PPy grafts via CH groups to poly-DND and hydrogen-bond interaction of the grafted PPy with surrounding CO groups results in the suitable alignment of energy levels of PPy and poly-DND and the spatial separation of HOMO on PPy and LUMO on poly-DND. This all is favorable for charge carriers separation between PPy and poly-DND.

**Figure 8:**
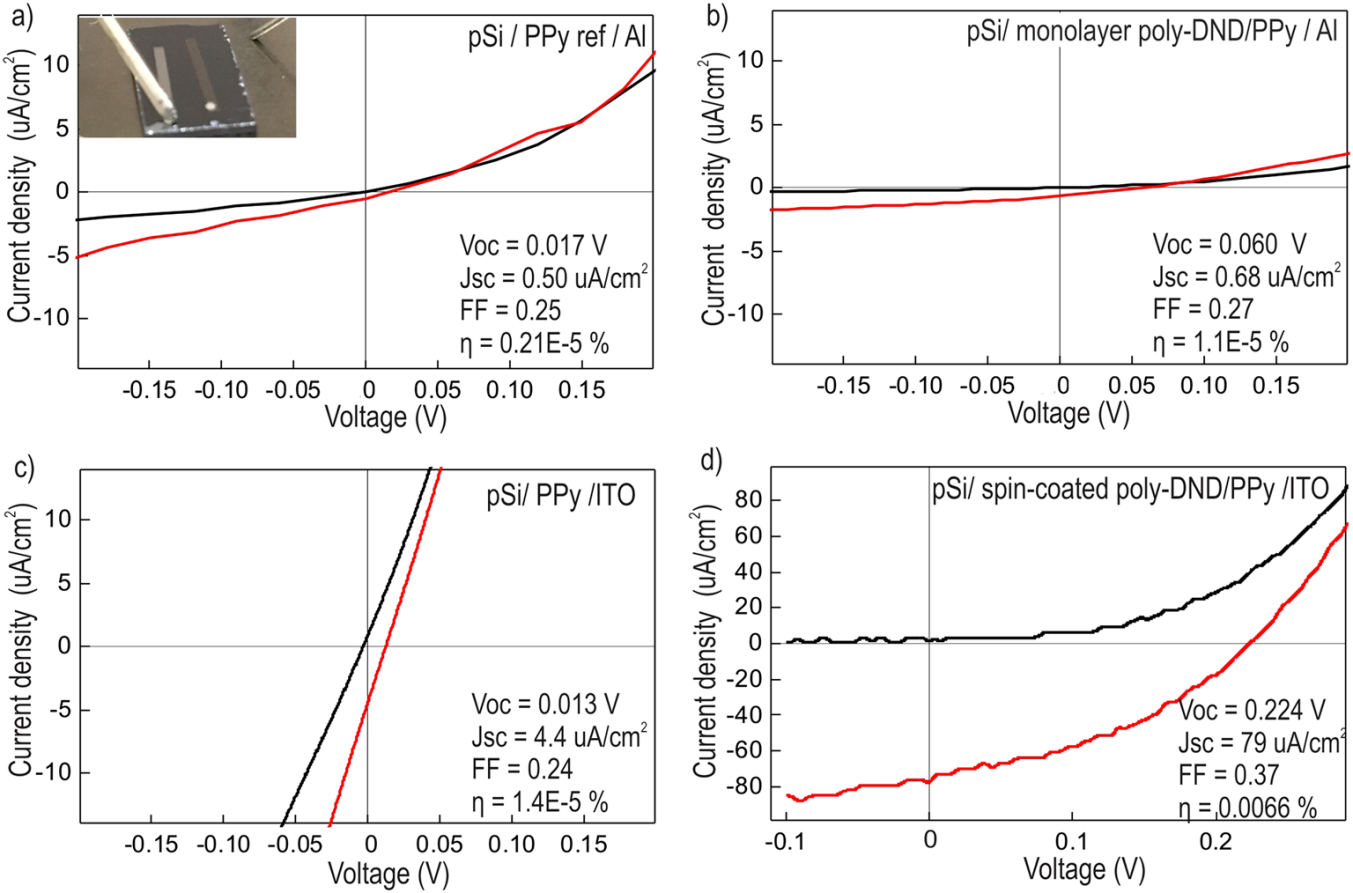
IV curves in the dark (black) and under solar simulator irradiation (red) of the solar cell with (**a**) pristine PPy as active material and Al as the top electrode; the inset picture shows the test solar cell with connected microelectrodes for IV characterization. (**b**) poly-DND/PPy composite as an active material with poly-DND monolayer and semitransparent Al layer as a top electrode. (**c**) pristine PPy as active material and ITO as the top electrode. (**d**) poly-DND/PPy composite as active material with 20 ± 2 nm thick poly-DND layer and ITO as the top electrode.

The composite with the most pronounced optoelectronic response (poly-DND/PPy) and PPy reference were embedded in the test solar cells to check the electronic transport characteristics. We show so far the best performing configurations based on a monolayer and spin-coated layer of DNDs and compare the role of Al or ITO as electrode materials as examples. Figure 8a shows IV characteristics in the dark and under illumination and the performance parameters of the solar cells with embedded PPy and Al as a top electrode. The inset image of Fig. 8a shows how the IV characteristics of the solar cells were measured. The performance parameters of the solar cell with the top Al electrode are enhanced after grafting PPy to a monolayer of poly-DND (Fig. 8b) compared to PPy in the reference solar cell. Power conversion efficiency (η) in poly-DND/PPy SC is 5 times higher compared to the neat PPy solar cell. This indicates that DND having large surface-to-volume ration carry more PPy than the bare Si substrate, thus absorbing more light and increasing the short-circuit current (I_sc_). Also, poly-DND facilitate the exciton dissociation in PPy and built-in voltage formation, improving the open-circuit voltage (V_oc_) in poly-DND/PPy composite solar cell compared to PPy solar cell.

When ITO is used as the top electrode, the short-circuit current (I_sc_) in PPy reference solar cell is increased about 9 times resulting in the power conversion efficiency enhancement of almost 7 times compared to PPy with Al top electrode (Fig. 8c). The reason for the performance improvement can be better transparency of ITO in visible light range compared to semitransparent Al and better electronic level alignment.

To investigate a way for possible further improvement of the solar cell performance with the poly-DND/PPy composite, we employed a thicker active layer by using the spin-coating procedure. Spin-coating was used to cast 38 ± 3 nm layer of poly-DND on silicon (Figure S6). After Py processing, the poly-DND layer thickness decreased to 20 ± 2 nm layer (due to desorption of DND into polymerization medium) with 6 ± 3 nm of PPy on top. We expected to address several issues with the spin-coating procedure, namely (a) more light can be absorbed by an increased amount of the material, (b) pinholes in the sample layer are avoided, (c) continuous pathways in electron donor and electron acceptor materials are created that improves the charge transport to the electrodes. Figure 8d shows the improved characteristics of the solar cell with the thicker active layer. Indeed, the short-circuit current in such solar cell increased to 79 μA/cm^2^. It is an order of magnitude higher than in a monolayer composite (Fig. 8b) PPy on the bare substrate, I_sc_ = 4.4 μA/cm^2^ (Fig. 8c). It is most likely due to the higher amount of light-harvesting PPy and fewer pinholes with better charge collection. Open-circuit voltage (V_oc_) increased by order of magnitude in spin-coated poly-DND/PPy composite (V_oc_ = 0.224 V) compared to the reference solar cell with the neat PPy (V_oc_ = 0.013 V), which shows that poly-DND has a favorable role in exciton dissociation and built-in voltage generation at the poly-DND/PPy junction. The efficiency increased several orders of magnitude in the spin-coated poly-DND/PPy solar cell (η = 6.6 × 10^−3^%) compared to PPy on the bare Si (η = 1.4 × 10^−5^%) as well as compared to the monolayer poly-DND/PPy composite (η = 1.1 × 10^−5^%). Thus, the test solar cell with poly-DND/PPy composite demonstrates a proof-of-principle and shows opportunities for further optimizations (by variation of materials for top/back electrode, finding the optimum thickness of the nanocomposite, morphology optimization, etc.).

## Conclusions

The present work showed that detonation nanodiamonds with various surface chemical groups can control assembly as well as the optoelectronic performance of nanocomposite with polypyrrole. We demonstrated layer-by-layer fabrication technology that is suitable for solar cell fabrication. Formation of the nanocomposite and pronounced interaction between polypyrrole and nanodiamond with the controlled alignment of the polymer chains by nanodiamond surface chemistry in such composite were evidenced by changes and appearance of vibrational bands in infrared spectroscopy performed both macroscopically and locally with nanoscale resolution and supported by atomic force microscopy. Moreover, AFM indicated that DNDs with different surface terminations assemble PPy oligomers in different ways. Different PPy alignments relative to DNDs surface might contribute to the different light-harvesting effects of the composites. The study of optical absorption and photovoltage generation revealed that the nanocomposite exhibits a highly enhanced absorption coefficient. The optical absorption of the composite is broad, covering well the visible spectrum from infrared to near UV (1–3.5 eV). Also, the photovoltage was enhanced compared to pristine materials. The positive polarity of SPV shows that DND acts as an electron acceptor and leave the positive charge on PPy molecules. Theoretical atomic-scale DFT calculations corroborated the experimental results and confirmed that electrons are indeed transferred from PPy into nanodiamond. Polyfunctional nanodiamond combining both oxygen and hydrogen surface groups appeared the most favorable for photovoltaics. The assembled test solar cells proved the functionality of the concept. Taking into account the general non-toxicity of nanodiamond, its chemical stability, and availability in large quantities, its applicability in organic photovoltaics is possible.

## Supplementary Information

Supplementary Information.
