# Author Correction: Nanodiamond surface chemistry controls assembly of polypyrrole and generation of photovoltage

**DOI:** 10.1038/s41598-021-87451-0

**Published:** 2021-04-09

**Authors:** Daria Miliaieva, Petra Matunova, Jan Cermak, Stepan Stehlik, Adrian Cernescu, Zdenek Remes, Pavla Stenclova, Martin Muller, Bohuslav Rezek

**Affiliations:** 1 Institute of Physics, Czech Academy of Sciences, Cukrovarnická 10, Prague 6, Czech Republic; 2 Faculty of Electrical Engineering, Czech Technical University in Prague, Technická 2, Prague 6, Czech Republic; 3 Attocube Systems AG, Eglfnger Weg 2, 85540 Munich, Germany

Correction to: *Scientific Reports* 10.1038/s41598-020-80438-3, published online 12 January 2021

The original version of this Article contained an error in the Acknowledgments section.

“The authors are thankful for P. Bauerova for assistance with SEM and solar cell fabrication. Helpful advice of E. Ukraintsev and J. Kocka are also gratefully appreciated. This work has been supported by the Czech Science Foundation project 15-01809S (GACR), student project SGS18/179/OHK4/3T/13 (CVUT) and the European Regional Development Fund project CZ.02.1.01/0.0/0.0/15 003/0000464 (CAP). It occurred in the frame of LNSM infrastructure. The copyright for the image of the house in Fig. 1b belongs to lanamaster@123rf.com.”

now reads:

“The authors are thankful for P. Bauerova for assistance with SEM and solar cell fabrication. Helpful advice of E. Ukraintsev and J. Kocka are also gratefully appreciated. This work has been supported by the Czech Science Foundation project 20-20991J (GACR), student project SGS18/179/OHK4/3T/13 (CVUT) and the European Regional Development Fund project CZ.02.1.01/0.0/0.0/15 003/0000464 (CAP). We also acknowledge use of the CzechNanoLab research infrastructure supported by the MEYS (LM2018110). The copyright for the image of the house in Fig. 1b belongs to lanamaster@123rf.com.”

The original Article has been corrected.

