# Supplementary material for: Nanodiamond surface chemistry controls assembly of polypyrrole and generation of photovoltage
Source: Sci Rep. 2021 Jan 12;11:590. doi: 10.1038/s41598-020-80438-3 (PMC7803993; doi:10.1038/s41598-020-80438-3)
Supplement: Supplementary file 1 — Supplementary Information. [file 41598_2020_80438_MOESM1_ESM.docx]

# **Supplementary Information for the article**

Nanodiamond surface chemistry controls assembly of polypyrrole and generation of photovoltage

Daria Miliaieva*^a,b^ Petra Matunova,^b^ Jan Cermak,^a^ Stepan Stehlik,^a^ Adrian Cernescu,^c^ Zdenek Remes,^a^ Pavla Stenclova,^a^ Martin Muller,^a^ and Bohuslav Rezek^b^

^a^Institute of Physics, Czech Academy of Sciences, Cukrovarnická 10, Prague 6, Czech Republic.

^b^Faculty of Electrical Engineering, Czech Technical University in Prague, Technická 2,
Prague 6, Czech Republic.

^c^Attocube systems AG, Eglfinger Weg 2, 85540 Munich-Haar, Germany.


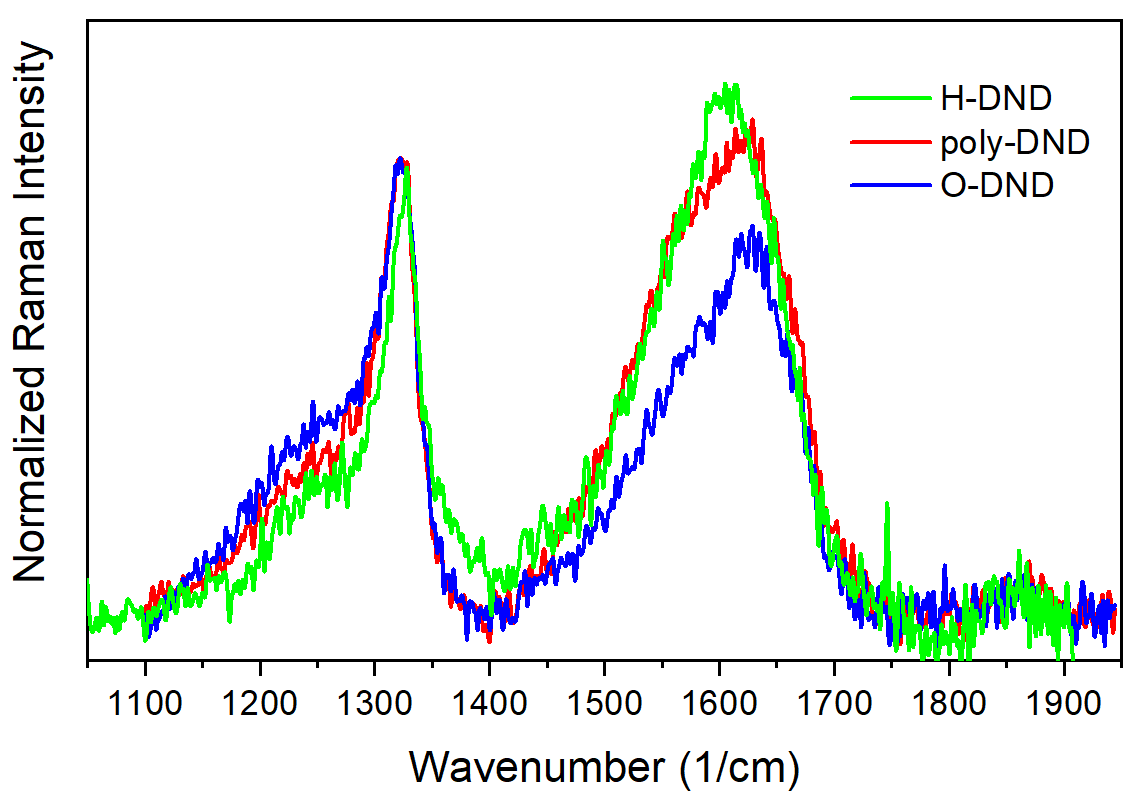


***Figure S1.*** *Raman spectra of H-DND, poly-DND and O-DND references.*


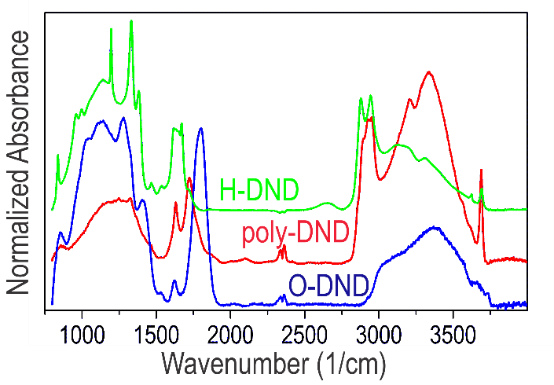


***Figure S2.*** *GAR-FTIR spectra of H-DND, poly-DND and O-DND references.*

| a) | poly-DND ref  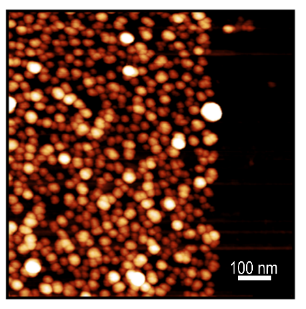 | poly-DND/PPy 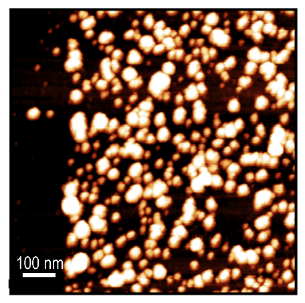 |
| --- | --- | --- |
|  | Z scale 12 nm | Z scale 12 nm |
| b) | H-DND ref  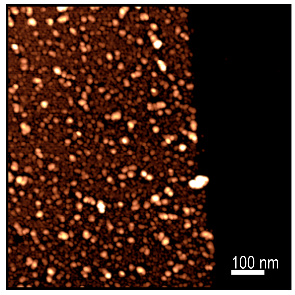 | H-DND/PPy  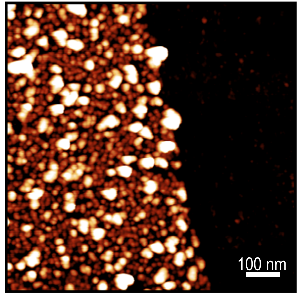 |
|  | Z scale 12 nm | Z scale 12 nm |
| c) | O-DND ref  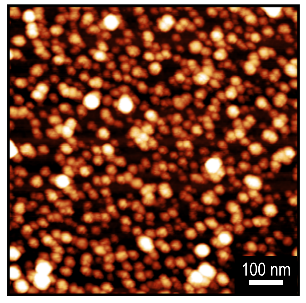 | O-DND/PPy  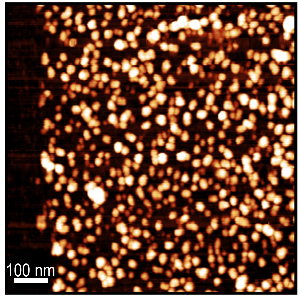 |
|  | Z scale 12 nm | Z scale 12 nm |

***Figure S3.*** *AFM morphology images of DND reference particles along with DND/PPy composites. The exposed substrate is a reference zero height.*


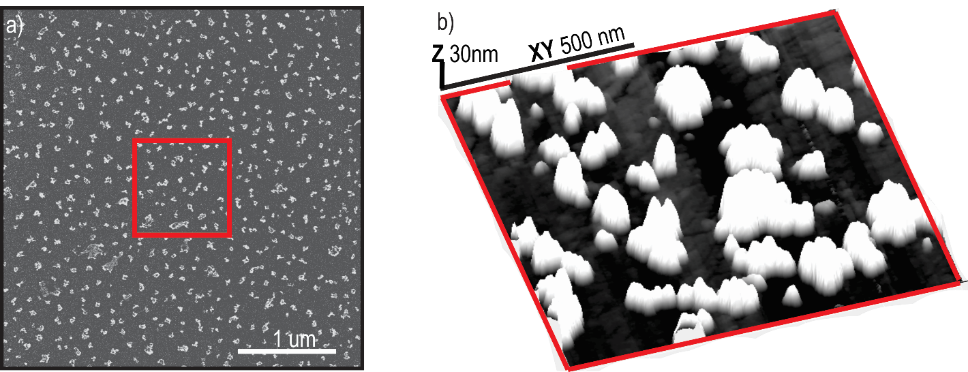


***Figure S4****. Low-density coverage of silicon substrate by poly-DND agglomerates (average size 30 nm) revealed by (****a****) SEM (****b****) AFM.*

| 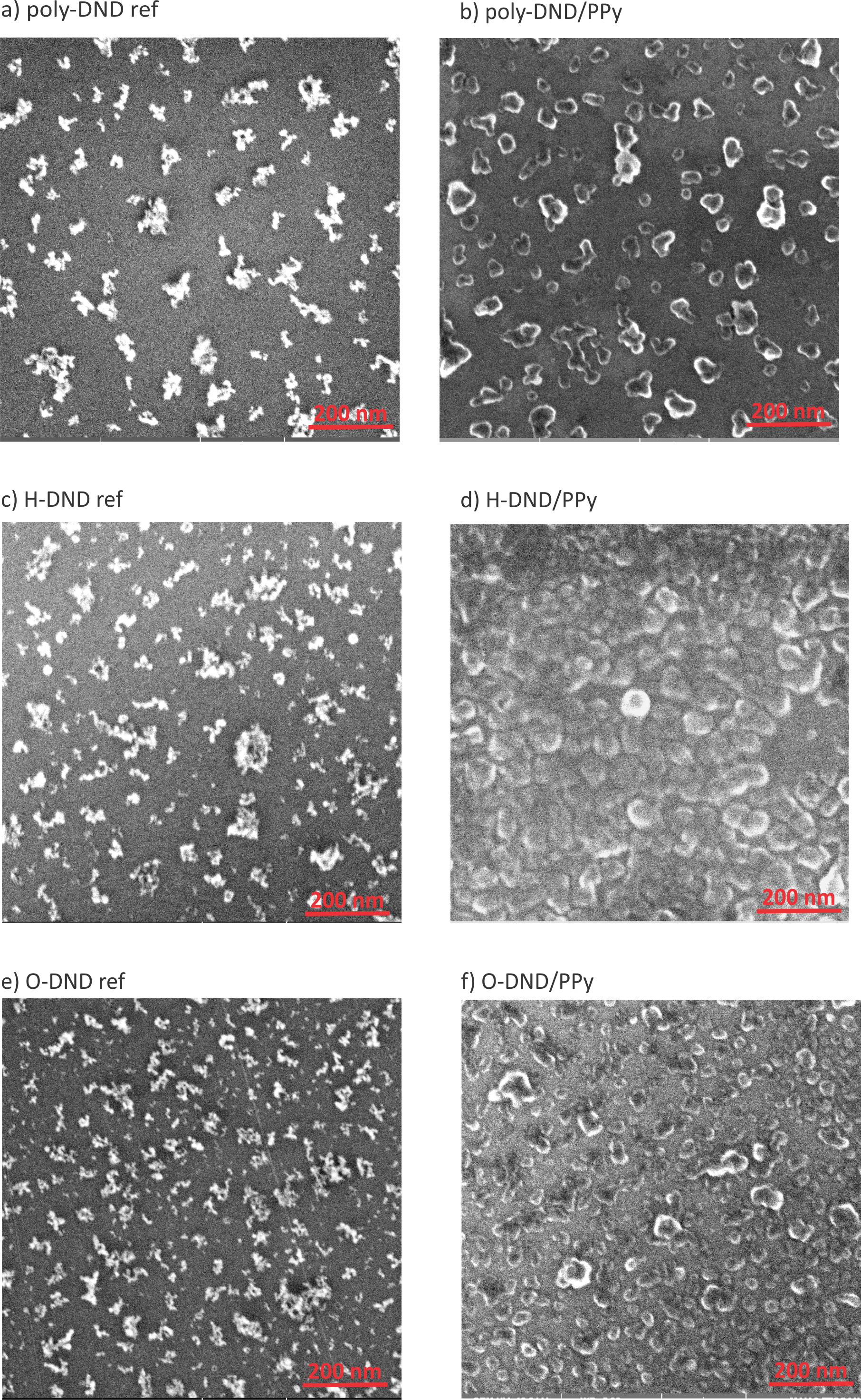 |
| --- |
| ***Figure S5****. SEM images of scattered DND agglomerates before (****a****,* ***c****,* ***e****) and after (****b****,* ***d****,* ***f****) polypyrrole grafting.* |

| 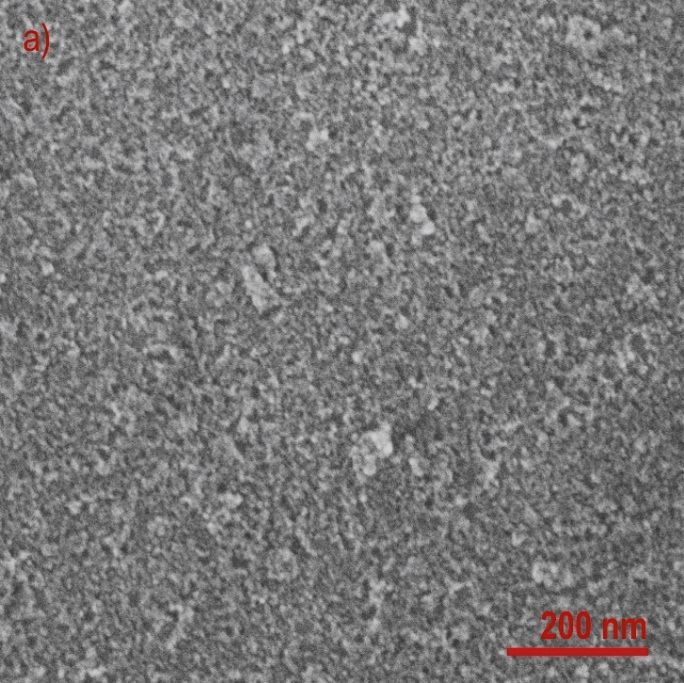 | 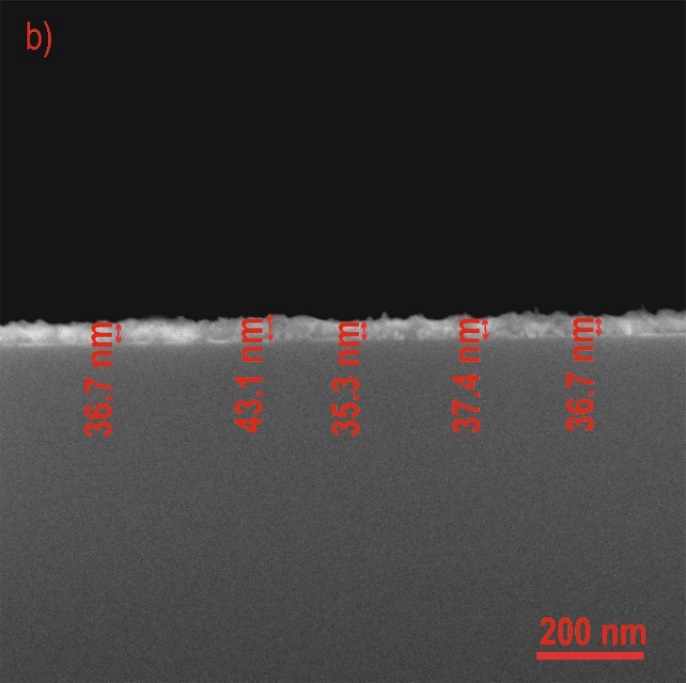 |
| --- | --- |
| ***Figure S6****. SEM image of the bare spin-coated poly-DND layer* ***a****) top view;* ***b****) cross-section for thickness determination. Average poly-DND ref thickness is 38 ± 3 nm.* | |

| 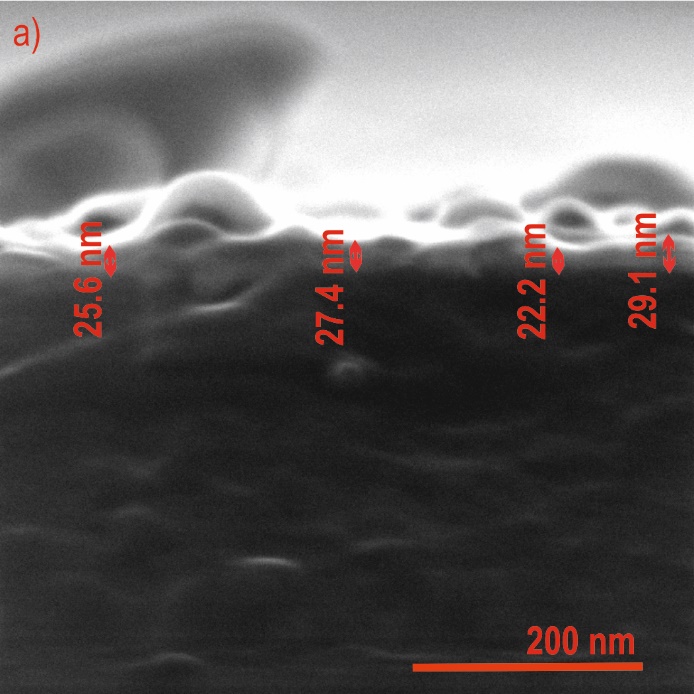 | 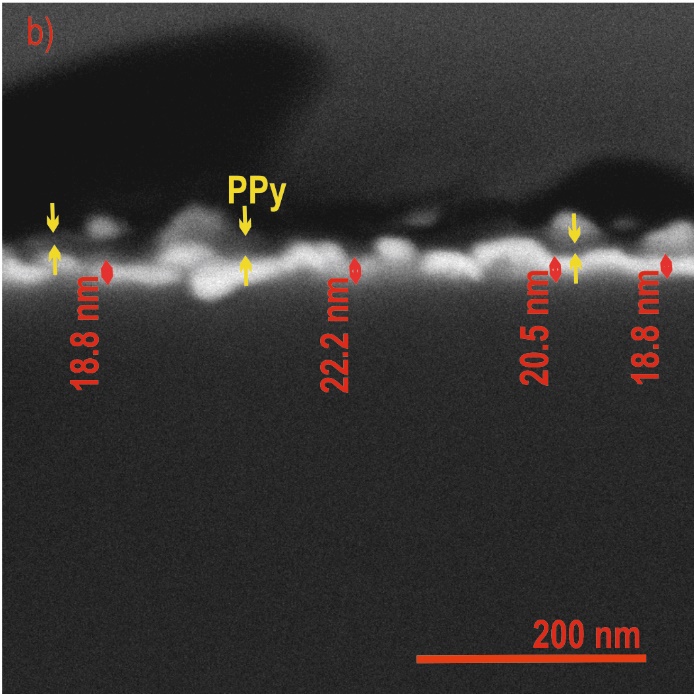 |
| --- | --- |
| ***Figure S7****. SEM images in* ***a****) secondary electrons (SE) and* ***b****) backscattered electrons (BSE) mode of the poly-DND/PPy composite layer cross-section. SE image reveals the overall composite thickness (26 ± 3 nm), while BSE image shows sp^3^ carbon of nanodiamond as a bright layer (average thickness 20 ± 2 nm) and sp^2^ carbon of PPy as blurred darker layer on top of the DNDs (average thickness 6 ± 3 nm).* | |

| 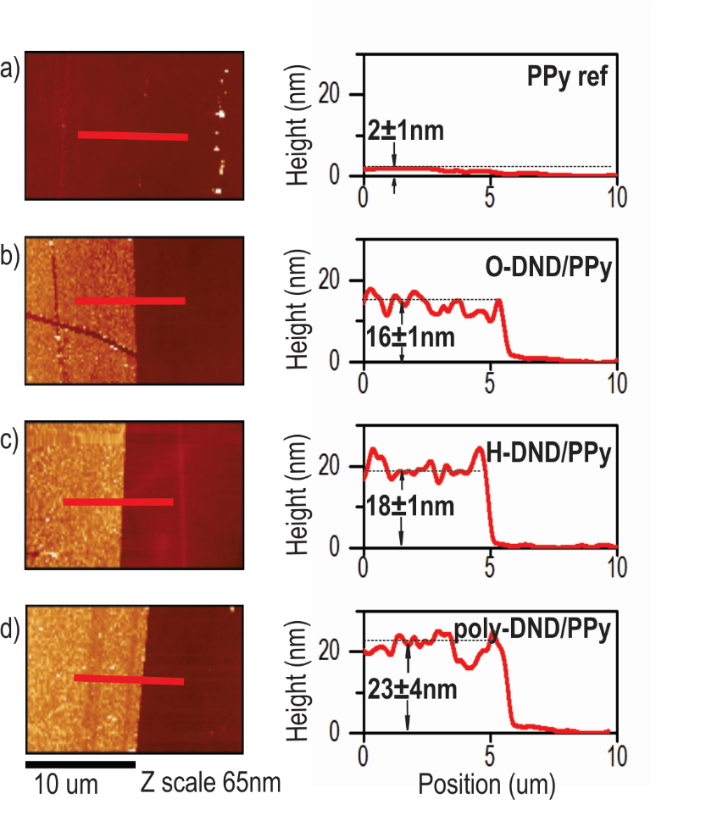 |
| --- |
| ***Figure S8.*** *AFM images of the samples on the border with an exposed bare glass substrate and corresponding height cross-sections of* ***a****) PPy reference* ***b****) O-DND/PPy* ***c****) H-DND/PPy* ***d****) poly-DND/PPy.* |

| 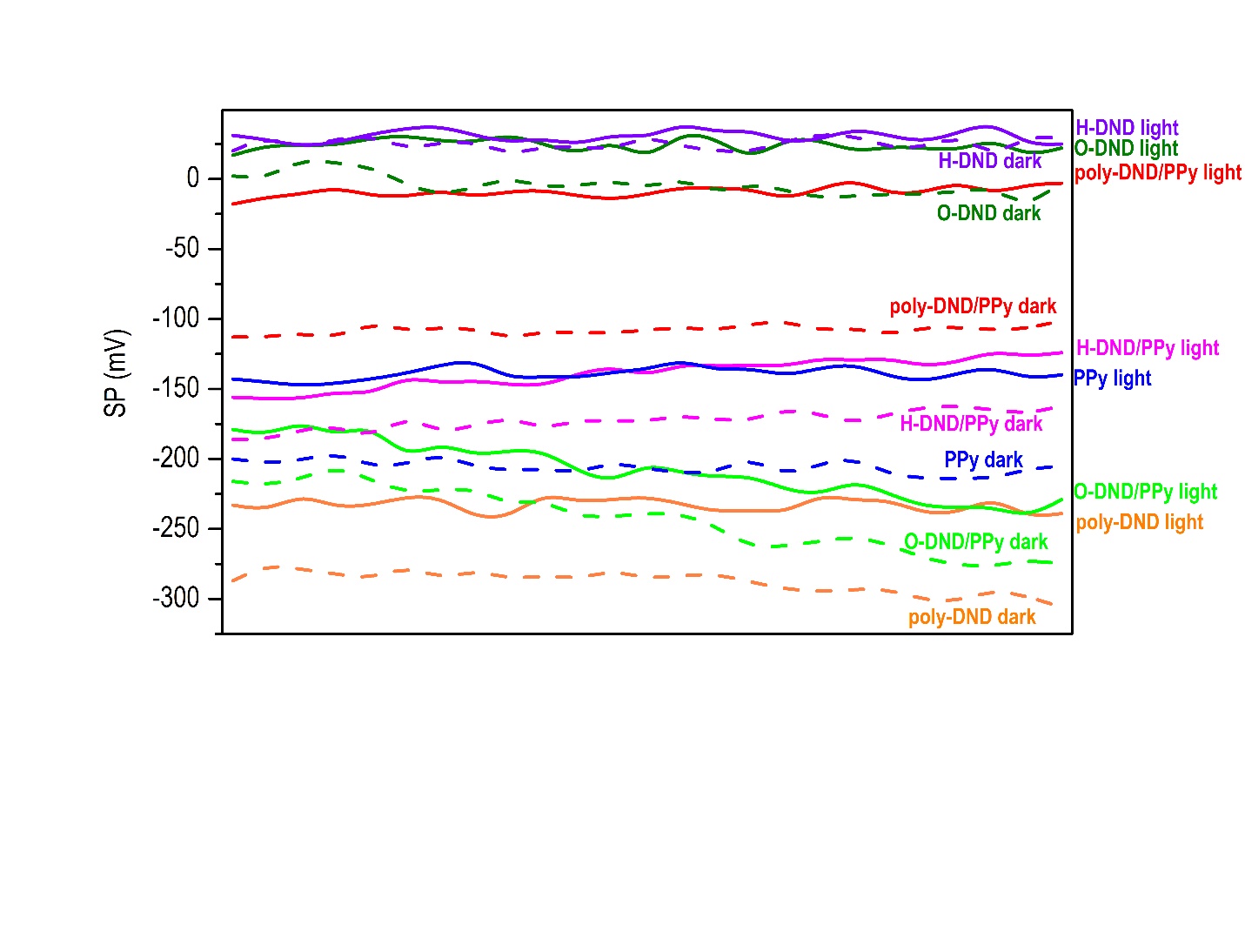 | | | | | |
| --- | --- | --- | --- | --- | --- |
| ***Figure S9.*** *Surface potentials of DND/PPy composites and their references in the dark and under illumination conditions.* | | | | | |
| ***Table S1.*** *Mean values of surface potential (SP) in the dark and under illumination and calculated surface photovoltage (SPV) of DND/PPy composites and their components.* | | | | | |
|  | SP dark (mV) | | SP light (mV) | | SPV (mV) |
| PPy | -206 ± 6 | | -139 ± 5 | | 67 ± 8 |
| poly-DND | -288 ± 8 | | -233 ± 6 | | 55 ± 9 |
| poly-DND/PPy | -108 ± 4 | | -9 ± 4 | | 99 ± 5 |
| O-DND | -4 ± 8 | | 24 ± 6 | | 28 ± 9 |
| O-DND/PPy | -243 ± 23 | | -208 ± 20 | | 35 ± 7 |
| H-DND | 25 ± 5 | | 30 ± 5 | | 6 ± 8 |
| H-DND/PPy | | -173 ± 7 | | -139 ± 11 | 34 ± 6 |
